# Validation of the Social Media Disorder Scale using network analysis in a large representative sample of Czech adolescents

**DOI:** 10.3389/fpubh.2022.907522

**Published:** 2022-08-22

**Authors:** Nika Šablatúrová, Karel Rečka, Lukas Blinka

**Affiliations:** Faculty of Social Studies, Masaryk University, Brno, Czechia

**Keywords:** problematic social media use (PSMU), social media addiction, validation, psychometrics, Network analysis, adolescents, Health Behavior in School-aged Children (HBSC)

## Abstract

**Background:**

The importance of studying the excessive use of social media in adolescents is increasing and so is the need for in-depth evaluations of the psychometric properties of the measurement tools. This study investigated the properties of the Social Media Disorder Scale (SMDS) in a large representative sample of Czech adolescents.

**Methods:**

We analyzed the representative sample of 13,377 Czech adolescents (50.9% boys), 11–16 years old, who participated in the Health Behavior in School-aged Children (HBSC) survey (2017–18), using confirmatory factor analysis (CFA) and network models. Furthermore, we evaluated the measurement invariance and constructed the validity of the SMDS.

**Results:**

We found support for a single dominant factor but not for strict unidimensionality. Several residual correlations were identified. The strongest were for: problems–conflicts–deceptions; persistence–escape; and preoccupation–tolerance–withdrawal. Girls, particularly 13- and 15-year-olds, scored higher than boys in the same age group, and 13- and 15-year-olds achieved higher scores than 11-year-olds, although some items were not invariant between the groups. The SMDS was positively related to other online activities, screen time, and falling asleep late, but negatively related to well-being and mental health.

**Discussion and conclusions:**

The SMDS showed solid psychometric properties and construct validity. However, small violations of measurement invariance were detected. Furthermore, the network analysis showed important residual relationships between the items.

## Introduction

The use of online social media, including social networks, has become an integral part of adolescent life. Despite some of its positives (1), a growing number of studies have shown potential negative impacts on mental health (2). Furthermore, excessive or problematic use of social media has been suggested to have characteristics similar to those of other behavioral addictions (3). Problematic social media use (PSMU) is a relatively novel concept, since pioneering studies were published only recently, after 2010 [see, e.g., (4)]. Moreover, PSMU is subject to controversies about its definition and measurement, and studies that investigate the validity of instruments measuring PSMU are limited. The absence of a clear definition and validated measurement tools that are based on a widely agreed set of diagnostic criteria hinder the progress of research.

In part, these issues could explain why PSMU has not yet been included in the Diagnostic and Statistical Manual of Mental Disorders [DSM-5; (5)] or the International Classification of Diseases [ICD-11; (6)]. According to the majority of studies, the general effect of social media use on mental health is small to moderate, depending on who is using it and how (7). PSMU has been demonstrated to be associated with various psychopathologies (8). Moreover, several systematic reviews already concluded that PSMU has a negative effect on various health-related outcomes in adolescents, such as depression or anxiety [e.g., (9–13)]. It was suggested that the ICD-11 could be accommodated to include PSMU. The category “Other specified disorders due to addictive behaviors” could be used to make a formal diagnosis (14). Although its current status remains controversial, PSMU can be considered a potential clinical disorder. As such, it is essential to address issues related to the assessment of PSMU and examine the tools that purport to measure it, their validity, and other psychometric properties. Therefore, the present study aims to improve the assessment of PSMU by analyzing one of its most popular measurement instruments, the Social Media Disorder Scale (SMDS), which is supposed to operationalize the clinical features of behavioral addictions (15).

There are several measurement tools to assess PSMU. Some scales focus on specific applications [e.g., Facebook; (16), or Instagram; (17)] or specific activities [e.g., taking selfies; (18)]. But scales that measure general PSMU have also been developed, such as the Problematic Social Networking Services Use Scale (19), the Social Media Addiction Test (20), and the Bergen Social Media Addiction Scale (21). This study investigates the validity of the nine-item SMDS (15). It measures nine addiction criteria (i.e., six core addiction criteria, two additional criteria that measure harmful consequences/problems and displacement, and a deception criterion). As such, the short scale represents a brief self-report questionnaire that covers each addiction criterion with one item; however, it also intends to measure one higher-order factor. In the original validation study (15), the scale demonstrated solid structural validity, a unidimensional (i.e., one-factor) model showed a good fit, and the internal consistency was satisfactory. Furthermore, strong correlations of the SMDS with compulsive internet use and self-reported social media addiction supported its convergent validity. Finally, weak to moderate correlations of the SMDS with depression, loneliness, impulsivity, the frequency of the daily use of social media, and attention deficit supported criterion validity (15).

The SMDS is one of the most widely used PSMU scales and it has been further validated by several studies in China (22), Indonesia (23), Nigeria (24), the Netherlands (25), and Turkey (26–28). Recently, Boer et al. (29) published a large cross-national validation study that involved 222,253 adolescents from 44 countries in North America and Europe (including the Czech Republic). The study reported good factorial validity for the scale in all of the countries, because the factor structure of the SMDS was deemed invariant across countries. Furthermore, measurement invariance for groups defined by gender and socioeconomic status was supported in all of the countries and for age groups in all of the countries except Malta. The positive association of the SMDS with the intensity of online communication, and its negative correlation with mental well-being, corroborated good criterion validity in almost all of the countries (29).

The present study aims to investigate the psychometric properties of the nine-item SMDS in a nationally representative adolescent sample from the Czech Republic. To achieve this, we evaluate the factor structure of the SMDS with a confirmatory factor analysis (a latent variable model). Furthermore, compared to previous studies, we newly investigate the residual correlations of SMDS items with a network approach, analyzing partial relations among items after the effects of other symptoms are controlled (i.e., network model) and after conditioning for both the effect of other symptoms and the effect of a latent variable (i.e., residual network model). These three approaches reflect three different explanations for the observed relations between items and symptoms (30). First, the latent variable approach views correlations among symptoms as “spurious”, caused by a latent variable (e.g., social media addiction). Second, the network approach does not assume a latent variable and views the relationships between symptoms as potential causal effects. For example, lying about the time spent on social media can cause arguments with parents, or vice versa. Third, the residual network approach assumes that a latent variable exists but does not rule out residual relations among symptoms, even after the common source of variance (i.e., a latent variable) is screened off. For example, social media disorder can cause both conflict with parents and the use of social media can be a coping strategy against negative emotions, but this does not rule out the fact that conflicts with parents can result in negative emotions, and, subsequently, negative emotions can trigger the use of social media as a means to cope with these emotions.

Furthermore, considering that gender and age are important factors in PSMU (31) and that problematic internet use increases with age during adolescence (32), we also examine whether the measurement model of the SMDS is invariant between gender and age groups. We also estimate the prevalence of PSMU in the current sample in boys and girls. In addition, we evaluate the construct validity of the SMDS with a structural model, examining latent correlations between the SMDS and theoretically related constructs, including psychological well-being, mental health, late sleep, and patterns of use of digital media (i.e., screen time, gaming time, frequency, preference for online communication). Compared to Boer et al. (29), who validated the SMDS with the same sample as the present study, we use stricter criteria to establish measurement invariance, explore the interaction between gender and age, employ a network model to explore relations among the SMDS items, and apply a structural model to assess the construct validity. Consequently, this study should provide more detailed information on the psychometric properties and validity of the SMDS in Czech adolescents. It should be noted that our study is largely exploratory. In particular, network analysis is a fundamentally exploratory technique to estimate key associations among symptoms. Thus, no hypotheses were generated.

## Methods

### Study design and participants

Data were collected in the Czech Republic in 2018 as part of the Health Behavior in School-aged Children (HBSC) study, which is a collaborative cross-national study of the World Health Organization (WHO). To obtain a representative sample of children aged 11–16, a multistage stratified design was applied: strata were defined by census regions and grades; schools were the primary sampling units. The schools were randomly selected from the list of all eligible schools in the Czech Republic. The list was obtained from the database of the Ministry of Education, Youth, and Sports of the Czech Republic. A total of 227 schools that were contacted agreed to participate in our survey, leading to a 97% response rate. Data were collected with standardized research protocols and followed the international standards of the HBSC study, which collaborated with the WHO in 2017 and 2018 to ensure the consistency of survey instruments, data collection, and processing procedures (33).

If schools agreed to participate, parents were informed about the study. They could approve or disapprove of their child's participation, and the adolescents themselves could decline to participate, even if their parents had approved. Adolescents did not record their name anywhere and could discontinue their participation at any time or skip questions they did not want to answer for any reason. Thus, participation was fully voluntary, and the respondents were ensured anonymity and the confidentiality of their responses. Data were collected in classrooms with school computers and data collection was supervised in all schools by trained administrators. The participating adolescents spent one lesson (45 min) answering the questionnaire. Teachers were not present during the administration. As a result, a representative sample of 13,377 Czech adolescents from elementary schools and from the corresponding grades at eight-year grammar schools was obtained. The study was approved by the Institutional Research Ethics Committee of the Faculty of Physical Culture, Palacky University Olomouc, No. 9/2016, on 4 March 2016.

### Measures

#### The Social Media Disorder Scale

The SMDS (15) measures the six addiction criteria (i.e., *preoccupation, tolerance, withdrawal, escape, persistence*, and *conflict*) and two additional criteria that refer to the harmful consequences of PSMU: *displacement* (i.e., displacement of social or leisure activities by PSMU) and *problems* (i.e., experiencing difficulties in important life domains due to PSMU). The scale also includes *deception* (e.g., lying about the time spent using SM). These nine aspects are derived from the DSM-5 criteria for diagnosing Internet Gaming Disorder (5). The SMDS consists of nine statements that can be answered “Yes” or “No”. We coded the “No” answers as 0 and the “Yes” answers as 1. The questionnaire was introduced with the statement: “We are interested in your experiences with social media. The term social media refers to social network sites (e.g., Facebook, Instagram, Twitter) and instant messengers (e.g., WhatsApp, Snapchat, Facebook Messenger).” Subsequently, respondents were asked “During the past year, have you…”, followed by, for example, “... tried to spend less time on social media, but failed?” (*persistence*) or “…often used social media to escape from negative feelings?” (*escape*). Adolescents with six or more affirmative responses could be labeled as “problematic users” (25); however, no clinically verified cut-off scores have yet been established. In our study, the reliability (McDonald's) was ω = 0.77.

#### Demographic characteristics

Gender was assessed by asking the respondents if they were a boy or a girl. Age was calculated as the difference between the year plus the month of birth and the date of the evaluation. For the measurement invariance analysis, participants were assigned to three categories based on their age (11-, 13-, and 15-year-olds).

#### Mental health

The HBSC Brief Symptom Checklist is a four-item measure. Its validity and reliability was further explored in an adolescent sample the HBSC study (34). To measure mental health, adolescents were asked to rate the frequency of experienced psychological symptoms. In particular, how often they felt low, felt nervous, experienced irritability or bad temper, and had difficulty sleeping in the preceding previous 6 months. Answer options ranged from 1 = “about every day” to 5 = “rarely or never”. The reliability (McDonald's) was ω = 0.71.

#### Late sleep routine (i.e., going to sleep late)

The participants were asked two questions: first, when they go to bed on school days and second, when they go to bed on weekends or holidays. The options ranged from “9 p.m. or earlier” to “4 a.m. or later”. The reliability (McDonald's) was ω = 0.81.

#### Well-being

The WHO well-being scale, which consists of five items (35), was used. The WHO-5 is a short measure that asks about the subjective well-being of the respondents. This tool is suitable for children aged 9 and above. In WHO-5 respondents were asked how often in the previous 2 weeks they felt, for example, calm and relaxed, cheerful and in good spirits, or active and vigorous. Answer options ranged from 1 = “never” to 6 = “all the time”. The reliability (McDonald's) was ω = 0.91.

#### The frequency of online communication

A four-item measure, adapted from the EU Kids Online Survey, exploring the intensity of online communication of adolescents (36), was used. Adolescents were asked about the frequency of their online communication on social networks with close friends, friends within a larger social group, online friends they met through the internet, and other people (e.g., parents, siblings, classmates, teachers). Answer options ranged from 1 = “never / rarely” to 5 = “almost all the time” throughout the day. Additionally, the option “do not know / does not apply” was included. The reliability (McDonald's) was ω = 0.72.

#### Preference for online social interaction

A three-item scale adapted from the EU Kids Online Survey (37) was used. Respondents were asked about their approach to online communication. In particular, they were asked if they find it easier to, for example, talk about private things, talk about feelings on the internet compared to face-to-face interaction. Response options ranged from 1 = “strongly disagree” to 5 = “strongly agree”. The reliability (McDonald's) was ω = 0.92.

#### Computer screen time

Adolescents were asked two questions about their computer screen time. They were asked how much of their free time (apart from schoolwork) they usually spend on their computer, tablet, or smartphone using Twitter, Instagram, Messenger, Facebook, browsing the internet, or communicating online. They were asked about the amount of time for school days and weekends separately. Response options ranged from 1 = “never” to 9 = “seven or more hours a day”. McDonald's omega was ω = 0.93.

#### Gaming frequency

Respondents were asked two questions about their frequency of gaming. In particular, they were asked how much of their free time they usually spend playing games on a computer, tablet, smartphone, or other electronic devices (apart from movement and fitness games), separately, for school days and weekends. Response options ranged from 1 = “never” to 9 = “seven or more hours a day”. The reliability (McDonald's) was ω = 0.92.

### Statistical analysis

The proportion of missing-item responses ranged from 0.67% (gaming frequency, computer screen time) to 19% (well-being, frequency of online communication), depending on the instrument. Only 62% of the respondents answered all of the items; however, 81% of the respondents skipped only one or two items. To keep respondents with incomplete data in the analysis, we used multiple imputations of the missing data to create 10 imputed datasets. This procedure was implemented in R using the *mice* package (38). We used a fully conditional specification (i.e., iterative, stepwise imputation of missing values based on a predefined imputation model). The imputation models included gender, age, and the total scores of all of the instruments described in the Method section as predictors for item responses. In the case of dichotomous items (only SMDS), we used a logistic regression model, but in the case of items on other scales, which always included five or more response options, predictive mean matching was used. Each substantive model was estimated separately on individual datasets and then the estimates were pooled according to Rubin's Rules using the *lavaan* (39) and *semTools* packages (40). The results differed only marginally compared to the results obtained from the analysis of complete cases alone. Therefore, we report only the results based on the imputed datasets.

Since the SMDS was developed as a unidimensional scale (15), we used confirmatory factor analysis to assess the fit of a unidimensional (one-factor) model. To detect small but theoretically meaningful violations of unidimensionality and explore the relations among items, above and beyond the effect of the latent variable, we also analyzed the residual correlations with a residual network model of partial correlations (30). We used the *psychonetrics* package (41) to estimate the model.

We investigated the construct validity of the SMDS with a structural model to estimate latent correlations between the SMDS and other variables, including online activities (computer screen time, gaming frequency), online communication (frequency of online communication, preference for online social interaction), well-being, mental health, and late sleep routine.

Previous studies also suggested that gender and specific age groups are important factors in PSMU (31). Therefore, we investigated whether the SMDS measurement is invariant for boys and girls (*n*_boys_ = 6,808 and *n*_girls_ = 6,569) and for age groups (*n*_11yo_ = 4,380, *n*_13yo_= 4,654, *n*_15yo_= 4,343), following the current guidelines for measurement-invariance testing with categorical indicators (42, 43): First, we tested configural invariance by fitting a model with all of the parameters freely estimated across groups. Second, we tested metric invariance by fixing item loadings. Third, we fixed the item thresholds to test for scalar invariance. Finally, we fixed the latent means of the groups to verify whether they differed significantly. To identify the model, we fixed the latent mean of one group to 0 and the latent variance to 1; for the other groups, except for the configuration model, these parameters were freely estimated. We also fixed the variance of the latent response variables to 1 and their intercepts to 0. We considered the metric invariance to be supported if ΔRMSEA ≤ 0.05 and ΔCFI ≥ −0.004, and the scalar invariance and latent means invariance to be supported if ΔRMSEA ≤ 0.01 and ΔCFI ≥ −0.004, as recommended by Rutkowski and Svetina (42). If full measurement invariance was not supported, we tested for partial invariance, freeing the parameters with the largest modification indices, one by one (44), until the aforementioned conditions for measurement invariance were reached.

## Results

### Research sample

The sample consisted of 13,377 adolescents from the Czech Republic. In total, 50.9% were boys. Based on the sum score of the nine-item SMDS questionnaire and its recommended cut-off score [6 points or more; (25)], 4.49% of boys and 5.69% of girls could be classified as problematic social media users. Table 1 shows the proportion of respondents (before multiple imputation) who agreed with each item.

**Table 1:** Prevalence of PSMU symptoms.

**During the past year, have you..**.	**Item**	**%**
…regularly found that you can't think of anything else but the moment that you will be able to use social media again?	1. Preoccupation	16.1
…regularly felt dissatisfied because you wanted to spend more time on social media?	2. Tolerance	13.9
…often felt bad when you could not use social media?	3. Withdrawal	17.9
…tried to spend less time on social media, but failed?	4. Persistence	25.7
…regularly neglected other activities (e.g., hobbies, sport) because you wanted to use social media?	5. Displacement	13.1
…regularly had arguments with others because of your social media use?	6. Problem	14.9
…regularly lied to your parents or friends about the amount of time you spend on social media?	7. Deception	10.2
…often used social media to escape from negative feelings?	8. Escape	26.4
…had a serious conflict with your parents, brother(s), or sister(s) because of your social media use?	9. Conflict	12.2
**PSMU (six or more symptoms)**		5.1

N = 12,337.

### Confirmatory factor analysis

Due to the categorical nature of the SMDS items, we used the diagonally weighted least squares estimator (DWLS), which is suitable for estimating models with categorical observed variables (45). The hypothesized one-factor model showed a good fit for the data, χ^2^ (27, *N* = 13,377) = 505.36, SF = 0.683, *p* < 0.001, CFI = 0.971, RMSEA =0.036 [90% CI (0.034, 0.039)], and SRMR = 0.045. To identify the model, both the variance of the latent response variables and the variance of the latent factor were fixed at a value of 1. Table 2 shows the estimates of the model parameters. Furthermore, all items shared a substantial variance with the latent variable, as all factor loadings exceeded 0.50, ranging from 0.57 to 0.81 (Table 2). All item thresholds were positive, ranging from 0.62 to 1.20, which means that the participants were inclined to disagree with the items rather than agree, as expected.

**Table 2:** CFA model of the Social Media Disorder Scale: item loadings and thresholds.

**Items**	**Loadings**			**Thresholds**		
	**Λ**	**99.5% CI**		**T**	**99.5% CI**	
Preoccupation	0.75	0.72	0.78	0.97	0.94	1.01
Tolerance	0.81	0.79	0.83	1.06	1.02	1.10
Withdrawal	0.75	0.73	0.78	0.90	0.87	0.94
Persistence	0.57	0.54	0.61	0.64	0.61	0.67
Displacement	0.68	0.64	0.71	1.10	1.06	1.14
Problem	0.69	0.66	0.72	1.03	0.99	1.07
Deception	0.74	0.71	0.77	1.24	1.20	1.28
Escape	0.67	0.64	0.70	0.62	0.59	0.66
Conflict	0.74	0.71	0.77	1.14	1.10	1.19

N = 13,377. The mean of the latent factor was set at 0 and its variance at 1. Delta parameterization was used (the total variance of the latent response variables was fixed at 1, so the residual variance is equal to 1–λ^2^).

### Residual network model

Despite the good global fit of the model, small, but not negligible, residual correlations (| *r* | = 0.05 to 0.10) between items indicated a local misfit, which means that the model underestimates the relationships between some items. Consequently, these results suggest that, although one factor dominates responses to the SMDS items, the SMDS is not strictly unidimensional.

Using a partial correlation network model (30), we analyzed these residual relationships. The residual correlation matrix of the one-factor model (see above) was used as input to estimate the network model. For model selection, we used a stepwise model search algorithm implemented by the *psychonetrics* package (41), which consists of five steps: (1) evaluate all models in which an edge is removed and that has *p* > 0.005, or an edge is added that has a modification index with *p* < 0.005; (2) if none of these models improves the Bayesian information criterion (BIC), return to the previous model and stop the algorithm; (3) update the model that improved the BIC the most; (4) evaluate all other candidate models that improved the BIC; and (5) if none of these models improves the BIC, go to Step 1, else go to Step 3.

**Figure 1:**
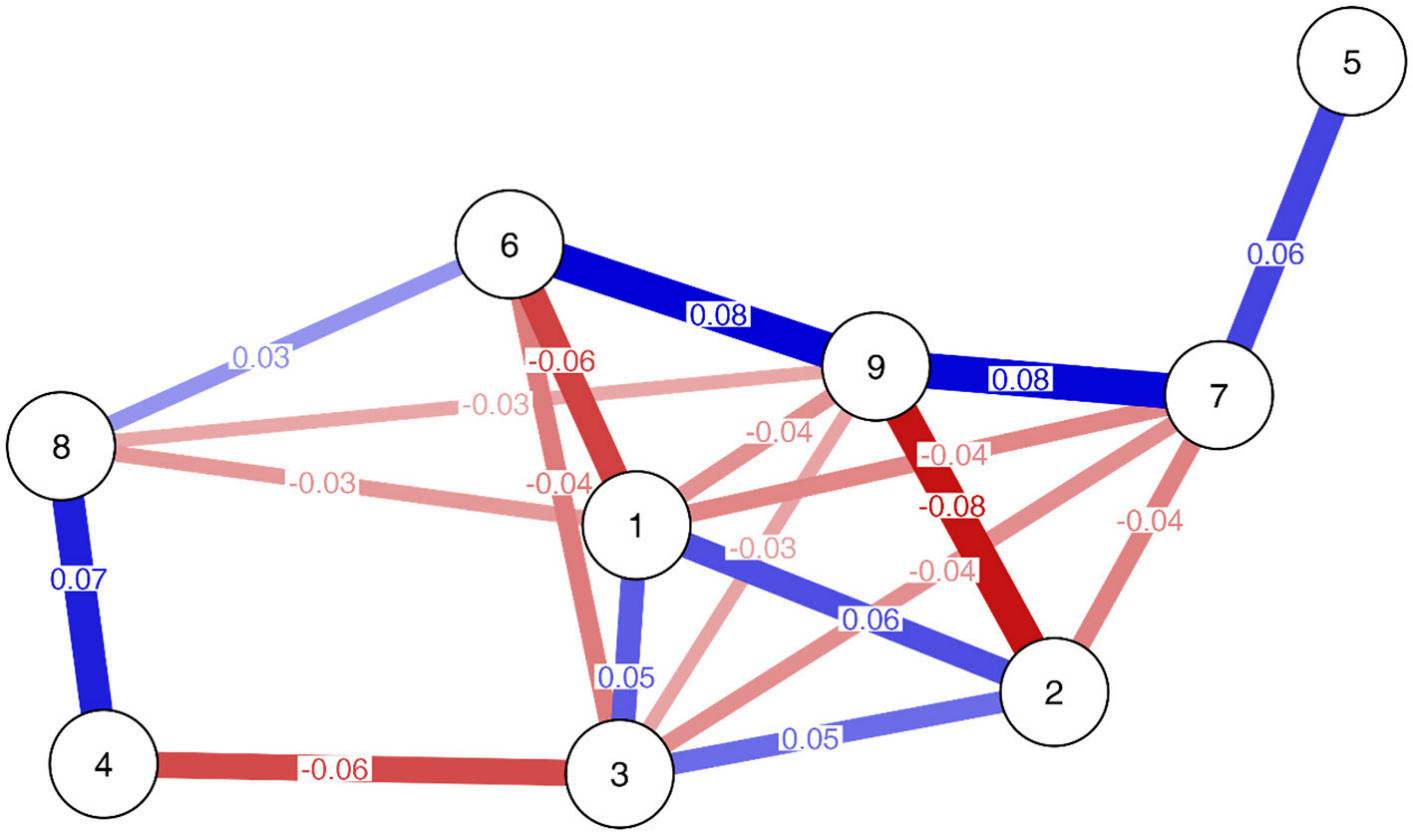
Residual network model of the Social Media Disorder Scale. Node labels indicate items: 1 = Preoccupation, 2 = Tolerance, 3 = Withdrawal, 4 = Persistence, 5 = Displacement, 6 = Problem, 7 = Deception, 8 = Escape, and 9 = Conflict. Red lines indicate negative edges, blue lines indicate positive edges. Color shades indicate edge weights (stronger edges are darker, weaker edges are lighter).

This algorithm led to a model that retained 19 edges (out of 36 possible edges). The model showed a good fit, χ^2^ (17, *N* = 13,377) = 42.33, *p* < 0.001, CFI = 0.970, RMSEA = 0.011 [90% CI (0.007, 0.015)], and SRMR = 0.009. The structure of the model is shown in Figure 1. It should be noted that there are partial correlations among item residuals; that is, conditional relationships exist between the items after conditioning on the latent variable and the effects of the other items. The strongest relations were observed between Item 6 (*problem)* and Item 9 (*conflict)*, and between Item 9 *(conflict)* and Item 7 (*deception*). Another stronger connection was observed between Item 4 (*persistence)* and Item 8 (*escape)*. A relationship was also observed between Item 6 *(problem)* and Item 8 (*escape)*. Furthermore, Item 5 (*displacement)* was the only item that had no partial relation to any other item, except Item 7 (*deception)*.

Item 1 (p*reoccupation*), Item 2 (*tolerance)*, and Item 3 (*withdrawal*) showed a positive partial residual correlation. However, they had negative associations with other items, particularly those related to interpersonal relations (i.e., Items 6, 7, 8, and 9). Lastly, the residuals of Item 3 (*withdrawal*) and Item 4 (*persistence*) showed a negative relationship.

There were three different groups of items whose residuals were positively correlated (but in most cases they were negatively correlated with residuals from other item groups). Analyzing the content of the items (see Table 1), Items 1, 2, and 3 (*preoccupation, tolerance*, and *withdrawal*, respectively) refer primarily to thoughts and feelings about social media use (i.e., cognitive-affective symptoms); Items 5, 6, 7, and 9 (*displacement, problem, deception*, and *conflict*, respectively) refer to the negative consequences of PSMU; and Items 4 (*persistence*) and 8 (*escape)* refer to the intentional use or avoidance.

### Network model

To estimate a standard network model, we used the same algorithm from the *psychonetrics* package (41) as described in the previous section. However, we used raw data as the input and the Ising model (defined on the domain {0, 1}), which is suitable for estimating pairwise interactions between binary variables (46). This model includes two main parameters: the threshold parameter reflects the tendency of a symptom (more generally, a node) to be active when all other symptoms are absent; and the interaction parameter reflects the tendency of two symptoms to become active together, relative to other states (i.e., one or both symptoms being inactive), and corresponds to edges with different weights, depending on its value. For each node, we also computed its strength, closeness, and betweenness centrality (47). Strength centrality is simply the sum of the absolute edge weights of a node; high strength implies that a node has (many) strong relations to other nodes. Closeness centrality is calculated as the inverse of the sum of all of the distances from a node to every other node; high closeness means that a node can “reach” other nodes relatively quickly. The betweenness centrality quantifies how often a node lies on the shortest path between two other nodes; high betweenness implies that a node can frequently “funnel” the activity between other nodes.

**Figure 2:**
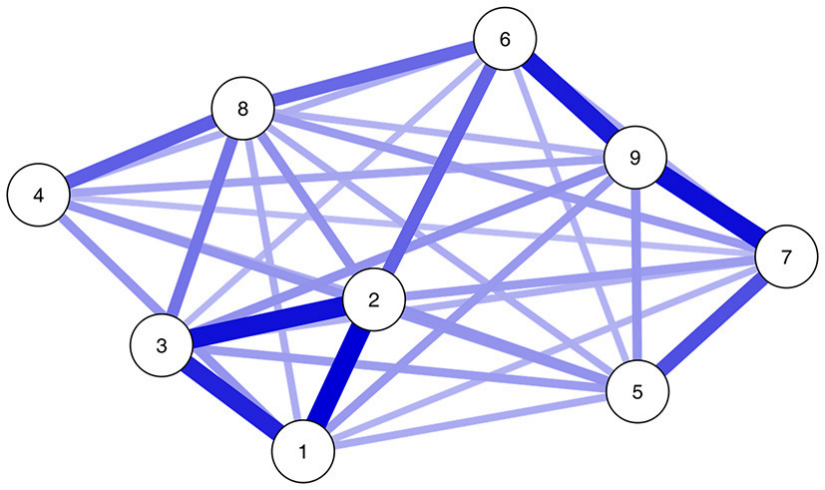
Network model of the Social Media Disorder Scale. Node labels indicate items: 1 = Preoccupation, 2 = Tolerance, 3 = Withdrawal, 4 = Persistence, 5 = Displacement, 6 = Problem, 7 = Deception, 8 = Escape, and 9 = Conflict. Blue lines indicate positive edges (all edges all positive). Color shades indicate edge weights (stronger edges are darker, weaker edges are lighter).

**Figure 3:**
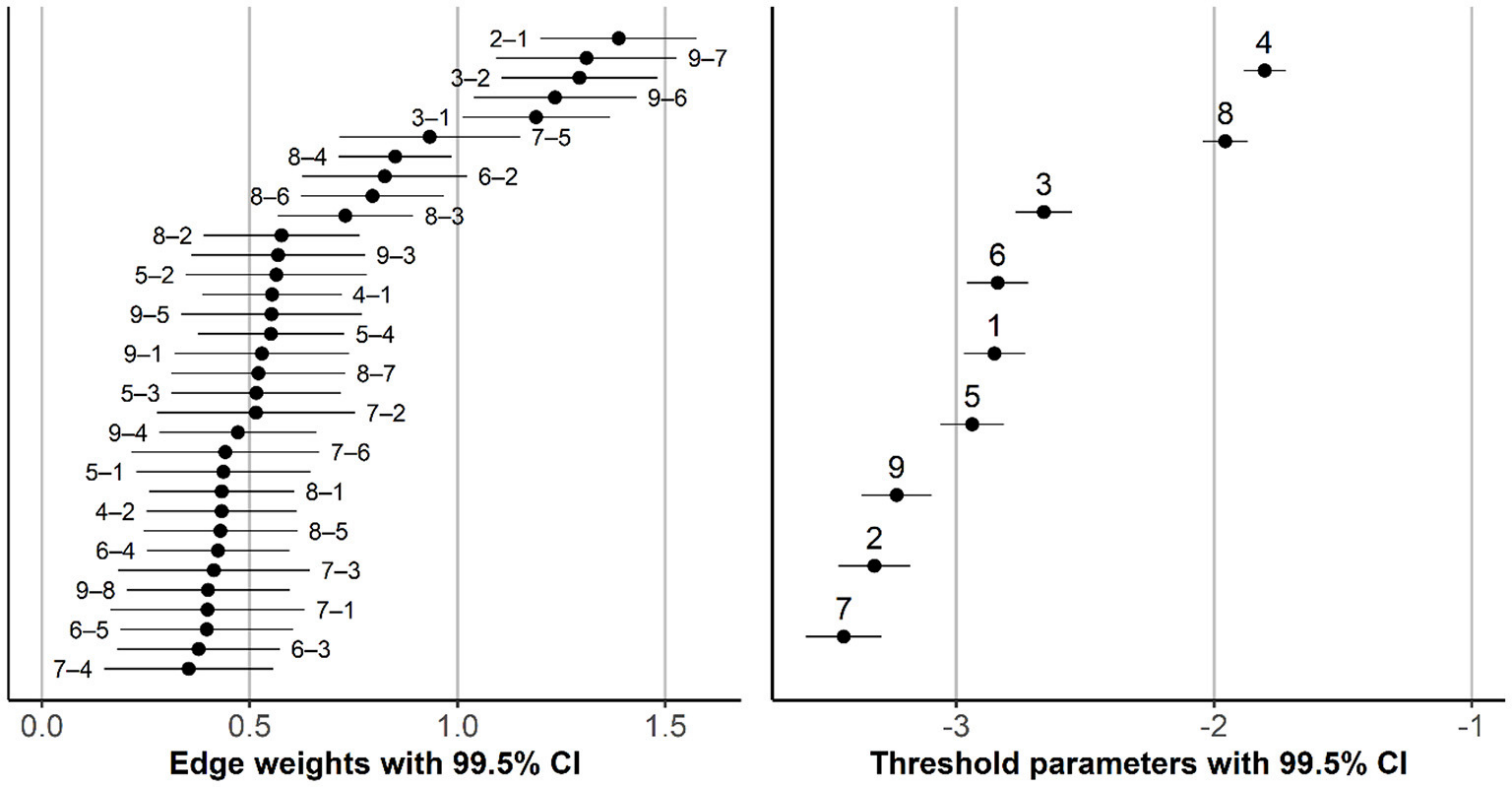
Edge weights (interaction parameters) and thresholds, with confidence intervals. Numbers in the plots indicate items: 1 = Preoccupation, 2 = Tolerance, 3 = Withdrawal, 4 = Persistence, 5 = Displacement, 6 = Problem, 7 = Deception, 8 = Escape, and 9 = Conflict. Number pairs (in the left plot) indicate which items the edge connects (e.g., “2–1” references the edge between Item 2 and Item 1).

The resulting network model was densely connected. Only three edges were omitted (between *preoccupation* and *problem*, between *tolerance* and *conflict*, and between *withdrawal* and *persistence*). In addition, all edge weights were positive; that is, the symptoms tended to support rather than hinder one another. Figure 2 shows the plot of the network model, and Figure 3 shows the estimated edge weight (i.e., interaction parameters) and thresholds, with 99.5% confidence intervals. Nodes 1-2, 1-3, and 2-3 (*preoccupation, tolerance*, and *withdrawal*, respectively), Nodes 6-9 (*problem* and *conflict*, respectively), and Nodes 7-9 (*deception* and *conflict*, respectively) were connected most strongly (i.e., highest edge weighs or interaction parameters), implying that these symptoms were most likely to co-occur.

**Figure 4:**
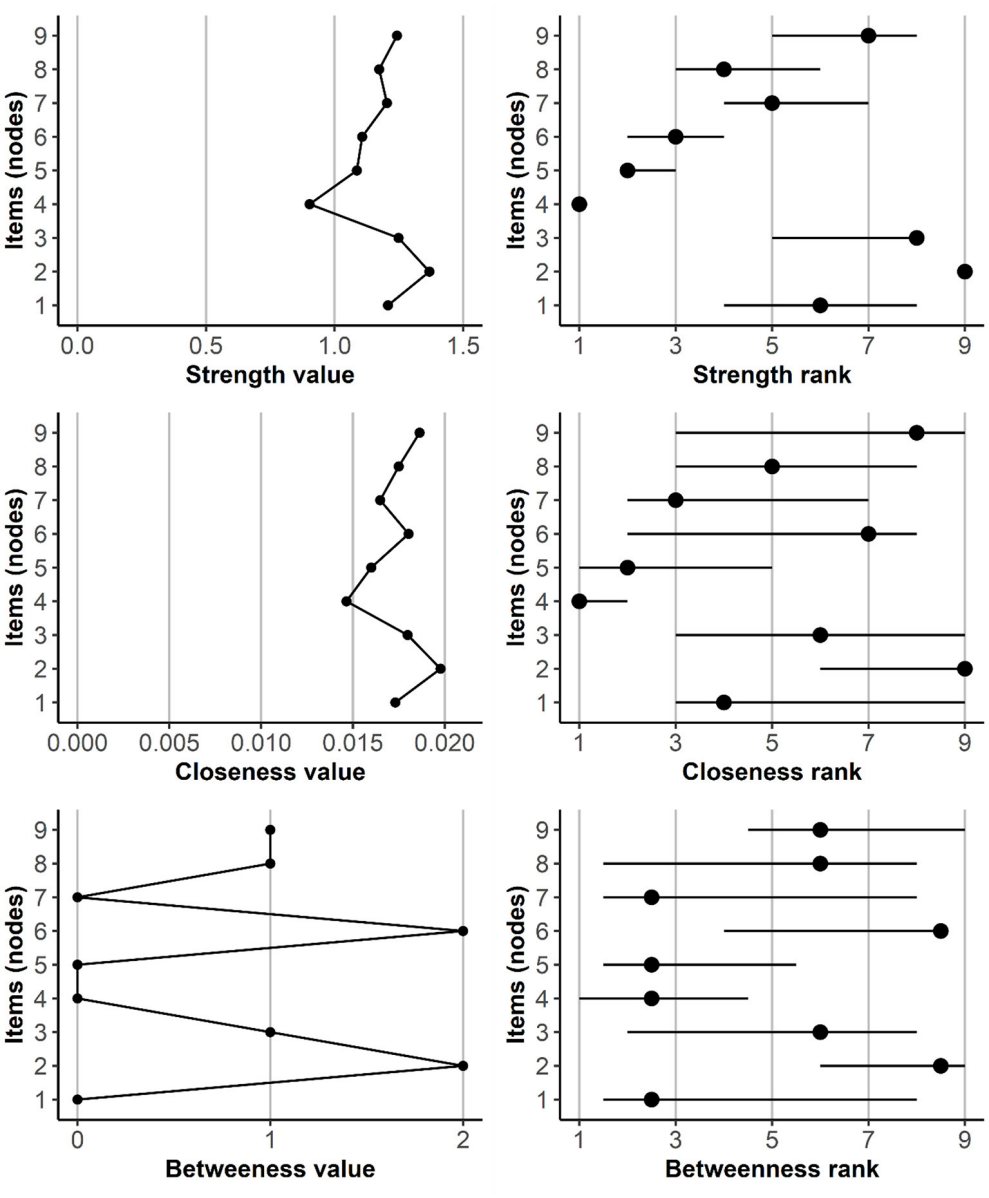
Values of centrality indices and node ranks, with 99.5% confidence intervals. Item (node) numbers indicate: 1 = Preoccupation, 2 = Tolerance, 3 = Withdrawal, 4 = Persistence, 5 = Displacement, 6 = Problem, 7 = Deception, 8 = Escape, and 9 = Conflict.

Nodes 4 and 8 (*persistence* and *escape*, respectively) had the highest thresholds, which means that they have the strongest propensity to become active, even when other nodes are inactive. Figure 4 shows the values of several centrality indices. In order to assess the stability of the ordering of the items according to their centrality and the differences between the items in their centrality, we performed a simple percentile bootstrap with 10,000 replications. For each bootstrap sample, we estimated the model with the same structure as our final model (i.e., with only three edges omitted; see Figure 2), calculated the centrality indices for each node, transformed them into ranks, and created 99.5% confidence intervals for these ranks. For any pair of items, non-overlapping confidence intervals imply a statistically significant difference in a given centrality index. As Figure 4 shows, the ordering of items according to their centrality index was rather unstable, especially for closeness and betweenness centrality. However, Node 2 (*tolerance*) seemed the most central and Node 4 (*persistence*) the least central across different indices. Furthermore, the connections of Nodes 2 (*tolerance*) and 6 (*problem*) appeared to provide the shortest path between “cognitive” and “negative consequences” symptoms.

### Measurement invariance

#### Measurement invariance between boys and girls

It was not necessary to free any item loadings to achieve the metric invariance between boys and girls (Table 3). To achieve (partial) scalar invariance, we had to free thresholds for *tolerance, persistence*, and *escape* items. Note that a higher threshold means a lower probability for agreeing with the item for the respondents from different groups but with the latent variable at the same level. *Tolerance* had a higher threshold for girls [τ = 1.21, 99.5% CI (1.14, 1.27)] compared to boys [τ = 1.09, 99.5% CI (1.03, 1.14)], but the *persistence* threshold was higher for boys [τ = 0.82, 99.5% CI (0.77, 0.87)] compared to girls [τ = 0.59, 99.5% CI (0.54, 0.65)]. The same was true for *escape*, which had a higher threshold for boys [τ = 0.80, 99.5% CI (0.75, 0.85)] compared to girls [τ = 0.60, 99.5% CI (0.55, 0.66)].

**Table 3:** Measurements of the invariance of the Social Media Disorder Scale.

**Groups by**	**Model**	**χ^2^** **(*df*)**	**SF**	**CFI**	**RMSEA** **[90% CI]**	**SRMR**	**Model** **comp**.	***Δχ*^2^** **(Δ*df*)**	**ΔCFI**	**ΔRMSEA**	**ΔSRMR**	**Decision**
Sex	M1 CI	581,91^***^ (54)	0,686	0.956	0.038 [0.035, 0.041]	0.048						
	M2 MI	565,03^***^ (62)	0,749	0.958	0.035 [0.032, 0.037]	0.050	M1 CI	34.25^***^ (8)	0.002	−0.003	0.002	Accept
	M3a SI	810,41^***^ (70)	0,729	0.938	0.040 [0.037, 0.042]	0.050	M2 MI	178.35^***^ (8)	−0.020	0.005	0.000	Reject
	M3b SI	585,89^***^ (67)	0,735	0.957	0.034 [0.032, 0.037]	0.050	M2 MI	10.97 (5)	−0.001	−0.001	0.000	Accept
	M4 LMI	782,98^***^ (68)	0,761	0.940	0.040 [0.037, 0.042]	0.050	M3 MI	164.13^***^ (1)	−0.016	0.006	0.000	Reject
Age	M1 CI	611,77^***^ (81)	0.691	0.968	0.038 [0.036, 0.041]	0.048						
	M2 MI	577,61^***^ (97)	0.801	0.971	0.033 [0.031, 0.036]	0.051	M1 CI	38.58^**^ (16)	0.003	−0.005	0.003	Accept
	M3a SI	841,09^***^ (113)	0.780	0.956	0.038 [0.036, 0.040]	0.051	M2 MI	194.62^***^ (16)	−0.015	0.005	0.000	Reject
	M3b SI	648,01^***^ (109)	0.784	0.968	0.033 [0.031, 0.036]	0.051	M2 MI	43.48^***^ (12)	−0.003	0.000	0.000	Accept
	M4 LMI	820,17^***^ (111)	0.833	0.957	0.038 [0.035, 0.040]	0.051	M3b SI	195.85^***^ (2)	−0.010	0.005	0.000	Reject
Sex	M1 CI	736,39^***^ (162)	0.697	0.972	0.040 [0.037, 0.043]	0.052						
and age	M2 MI	736,37^***^ (202)	0.873	0.974	0.034 [0.032, 0.037]	0.057	M1 CI	107.36^***^ (40)	0.002	−0.005	0.006	Accept
	M3 SI	829,54^***^ (233)	0.850	0.971	0.034 [0.031, 0.036]	0.057	M2 MI	69.27^***^ (31)	−0.003	−0.001	0.000	Accept
	M4 LMI	1,318.54^***^ (238)	0.917	0.948	0.045 [0.043, 0.048]	0.057	M3 SI	513.12^***^ (5)	−0.023	0.011	0.000	Reject

SF, scaling factor of the χ^2^ test statistic; CI, configural invariance; MI, metric invariance (fixed loadings); SI, scalar invariance (fixed thresholds); LMI, latent means invariance.

** p < 0.01,

*** p < 0.001.

After setting the latent means as equal for boys and girls, the fit of the model deteriorated significantly, Δχ^2^(1, *N* = 13,377) = 164.13, *p* < 0.001, ΔCFI = −0.016, ΔRMSEA = 0.006, and ΔSRMR = 0.000, suggesting that there are significant differences between boys and girls at the mean level for the symptoms of social media disorder. To estimate the difference, we used the model “Sex M3b SI” (see Table 3). For boys, the latent mean and variance were fixed to 0 and 1, respectively; for girls, these parameters were freely estimated. The latent mean for girls, *M* = 0.21 [99.5% CI (0.14, 0.27)], *SD* = 0.90 (99.5% CI [0.87, 0.94]) was higher than the latent mean of boys. However, the overall violation of measurement invariance was rather mild, as evidenced by the fact that, when all item loadings and thresholds were set to equality (model “Sex M3a SI”), the latent mean for girls was only slightly higher, *M* = 0.25 [99.5% CI (0.19, 0.32)], compared to model “Sex M3b SI”.

#### Measurement invariance across age groups

It was not necessary to free any loadings across age groups to achieve metric invariance. However, to achieve (partial) scalar invariance, the thresholds of *problem* and *escape* items were freed. For these items, the 11-year-old group had the highest threshold values, but the 15-year-old group was the lowest. Fixing the latent means to be identical across age groups significantly deteriorated the model fit, Δχ^2^ (2, *N* = 13,377) = 195.85, *p* < 0.001, ΔCFI = − 0.010, ΔRMSEA = 0.005, and ΔSRMR = 0.000. Therefore, the “Age M3b SI” model was used to estimate the difference between age groups. The latent mean and variance for 11-year-olds were fixed to 0 and 1, respectively, but for other age groups, these parameters were freely estimated. The latent mean for 13-year-olds was *M* = 0.24 [99.5% CI (0.15, 0.32)], *SD* = 0.99 [99.5% CI (0.94, 1.03)] and the latent mean for 15-year-olds was *M* = 0.21 [99.5% CI (0.13, 0.29)], *SD* = 0.97 [99.5% CI (0.93, 1.02)]. When fixing all loadings and thresholds (including *problem* and *escape* items) to equality across age groups (model “Age M3a SI”), the latent mean was *M* = 0.28 [99.5% CI (0.20, 0.36)] for 13-year-olds and *M* = 0.31 [99.5% CI (0.23, 0.39)] for 15-year-olds, which is slightly higher compared to model “Age M3b SI”. Therefore, we deem the measurement non-invariance across age groups to be mild, but not negligible.

#### Measurement invariance for gender and age simultaneously

**Figure 5:**
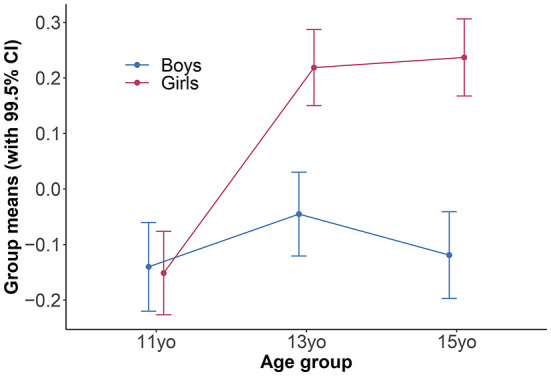
Latent means of the Social Media Disorder Scale, depending on gender and age.

Finally, we estimated a multigroup model where groups were defined by both gender and age (e.g., 11-year-old boys, 11-year-old girls). This resulted in six groups. The configural model fit well. It was not necessary to free any loadings between groups to achieve metric invariance. Based on previous results, we allowed the thresholds for the *tolerance* and *persistence* items to vary between boys and girls, the threshold for *problem* to vary between age groups, and the thresholds for *escape* to vary between both gender and age to achieve scalar invariance. This model allowed us to investigate the potential interaction between gender and age. We parametrized the model so that the average variance of latent variables equaled 1 and their grand mean equaled 0. As a result, the differences between the group means can be interpreted as Cohen's *d*. As shown in Figure 5, the difference between 11-year-old boys and girls was negligible, *d* = −0.01, 99.5% CI (−0.13, 0.11), but the difference between 13-year-old boys and girls was greater, *d* = 0.26, 99.5% CI (0.15, 0.38), as was the difference between 15-year-old boys and girls, *d* = 0.36, 99.5% CI (0.24, 0.47).

### Construct validity

The structural model that included the latent correlations of the SMDS with other relevant variables was used to verify the construct validity of the SMDS. In general, the model showed a good fit for the data, χ^2^ (347, *N* = 13,377) = 2,636.71, SF = 0.708, *p* < 0.001, CFI = 0.922, RMSEA = 0.022 [90% CI (0.021, 0.023)], and SRMR = 0.025.

The analysis revealed significant positive correlations of the SMDS with other online activities and falling asleep late, but negative correlations with well-being and mental health. The highest correlations were observed between the SMDS and the frequency of online communication, computer screen time, and mental health. All correlations between latent variables are reported in Table 4.

**Table 4:** Correlations among latent variables.

**Variables**	**1. SMDS**	**2. MH**	**3. OCF**	**4. OSP**	**5. CST**	**6. GF**	**7. SLP**	**8. WB**
1. SMDS		−0.16	0.48	0.29	−0.24	−0.40	0.37	−0.16
2. MH	−0.38 [−0.42, −0.35]		0.39	0.26	−0.29	−0.66	0.77	0.32
3. OCF	0.42 [0.39, 0.46]	−0.19 [−0.22, −0.15]		0.33	−0.17	0.42	0.08	0.76
4. OSP	0.29 [0.26, 0.32]	−0.21 [−0.24, −0.18]	0.25 [0.22, 0.28]		−0.39	0.22	0.20	−0.04
5. CST	0.38 [0.35, 0.41]	−0.26 [−0.29, −0.23]	0.52 [0.49, 0.54]	0.22 [0.19, 0.24]		0.19	0.17	0.25
6. GF	0.17 [0.14, 0.20]	−0.03 [−0.06, 0.00]	0.24 [0.21, 0.27]	0.12 [0.10, 0.15]	0.37 [0.35, 0.39]		0.07	−0.16
7. SLP	0.31 [0.28, 0.35]	−0.28 [−0.31, −0.25]	0.46 [0.43, 0.48]	0.24 [0.21, 0.27]	0.47 [0.45, 0.49]	0.31 [0.29, 0.34]		−0.45
8. WB	−0.28 [−0.32, −0.25]	0.50 [0.47, 0.52]	0.00 [−0.04, 0.03]	−0.15 [−0.18, −0.13]	−0.15 [−0.18, −0.13]	−0.04 [−0.06, −0.01]	−0.19 [−0.21, −0.16]	

Bivariate correlations with 99.5% confidence intervals (in brackets) are shown in the left triangle, and implied partial correlations are shown in the right triangle. SMDS, Social Media Disorder Scale; MH, mental health; OCF, frequency of online communication; OSP, preference for online social interaction; CST, computer screen time; GF, gaming frequency; SLP, going to sleep late; WB, well-being.

In summary, the associations were significant and in the expected directions: the higher the level of PSMU, the lower the level of well-being and mental health, but the higher the frequency of online activities and late sleep. A relatively low association was found between the SMDS and gaming, while all other correlations were found to be moderately strong. Thus, these results support the construct validity of the test.

## Discussion

The purpose of this study was to conduct a thorough investigation of the psychometric properties of the SMDS with a large representative sample of Czech adolescents. The scale was found to have solid psychometric properties with one dominant factor. However, strict unidimensionality was not achieved. A network analysis of residual correlations discovered several groupings of more similar items that can be interpreted as the “cognitive-affective” aspects of addiction, the “behavioral-consequential” and “persistence-avoidance” aspects of PSMU. Strict measurement invariance did not hold between boys and girls (13- and 15-year-old girls scored higher on symptoms) nor between age groups (13- and 15-year-olds scored higher than 11-year-olds). The degree of non-invariance between the groups was rather small but should not be ignored. Regarding the construct validity, the structural model revealed that the SMDS is moderately associated with the higher use of digital media (specifically screen time, frequency of and preference for online communication), late sleep patterns, and lower mental health and well-being.

The use of the sum score of the nine items is justified when the scale measures one underlying dimension to which all nine items contribute substantially. Furthermore, the symptoms of behavioral addictions are generally considered to be the consequences of a latent construct, which has been implicated in the case of Social Media Disorder (28, 48). However, Fung (22) suggested a two-factor structure with the *conflict, problem*, and *deception* items representing a separate latent factor. In our study, although the one-factor model showed a good fit for the data, strict unidimensionality was not supported. Our findings partially contrast with those of Boer et al. (25), Savci et al. (27), and Boer et al. (29), who argued that the scale is unidimensional and measures one latent variable.

Despite the good fit of the unidimensional model, the residual correlations among items indicated a local misfit. In other words, the model underestimated the associations among some symptoms. Therefore, to find meaningful relationships between items, we analyzed the residual relationships of SMDS items with a network model. In general, there were three slightly different groups of symptoms. *Preoccupation, tolerance*, and *withdrawal* items, which can be labeled as “cognitive-affective” symptoms, showed positive partial residual correlations. Although initially they were conceptualized as indicators for a common latent variable (15), In network modeling, symptoms are not considered to be the “passive consequences” of a common cause (latent variable-PSMU) but as independent elements with causal power that could mutually trigger each other (e.g., not being able to think of anything other than using social media can cause dissatisfaction when an individual is not allowed to do so, or vice versa) (49). Moreover, we believe that these three items are conceptually related and reflect the cognitive-affective aspect of addiction. Another group of symptoms—*problem, deception, conflict*, and *displacement—*is primarily related to the behavioral-consequential aspects of PSMU. These symptoms potentially reflect the consequences of preferring social media over other activities or relationships. However, the direction of this relationship could be reversed; that is, the use of social media could serve as a coping strategy in response to interpersonal conflicts and problems. Most likely, both directions could be present at the same time, because media effects and media use are usually in transactional relationships (i.e., mutually influence one another) (50).

A previous study (25) suggested the presence of six of the nine items (i.e., symptoms) as the cut-off score. This means that at least one behavioral-consequential symptom is always present; however, the cognitive-affective or persistence-avoidance symptoms could be missing, which may be problematic. It has been suggested that the presence of “cognitive-affective” symptoms may be considered harmful, even without a “behavioral-consequential” criterion (51). At the same time, it is important to avoid overpathologizing the behaviors that do not need intervention (52). Therefore, we suggest that a multiple factor solution should be considered, with the inclusion of all three of the aspects of addiction identified in this study. For example, to ensure their inclusion the cut-off should be at least eight of the nine items instead of six.

The strong relation between *persistence* and *escape* suggests that individuals who use digital media to relieve negative emotions may experience greater difficulties controlling their excessive use (53). However, it should be noted that, based on our results, these two symptoms were relatively common, and, therefore, they may lack specificity. This means that even “non-problematic” users may try to limit their time on social media and that the use of social media may sometimes represent a useful coping strategy. Furthermore, people may view using social media to escape negative feelings as a positive feature (54), corresponding to what Kardefelt-Winther (55) labeled as “compensatory internet use”, which is an alternative to the addiction model.

While *persistence* was one of the least central, *tolerance* was one of the most central symptoms. This is in line with another study (28) that also explored the network model of the SMDS and identified *tolerance*, together with *preoccupation* and *withdrawal*, as central symptoms. Interestingly, the very existence of withdrawal symptoms and tolerance has been doubted (52, 56), but our study suggests that, at least in problematic or excessive social media use, they play an important role. Furthermore, it has been argued that targeting and prioritizing central symptoms is clinically meaningful in the treatment of mental disorders (57). Therefore, our findings imply that *tolerance* deserves preferential attention when examining symptoms and designing clinical interventions and prevention efforts. Furthermore, the link between *tolerance* and *problems* provided the shortest path between the “cognitive-affective” and “behavioral-consequential” aspects. This may represent an *escalation*, since spending more time on social media may lead to various conflicts, and vice versa. These conflicts may lead to increasing time spent online. Since targeting important paths that bridge different groups of symptoms can improve the effectiveness of clinical intervention (58), this path is the prime candidate in this regard. However, we must also note that the symptoms in the network model were densely connected and tended to support each other.

Another important finding of the present study is that some item thresholds were not invariant across gender and age groups. The *tolerance* item was more popular for boys while *persistence* and *escape* were more popular for girls. In this context, more popularity means that members of one group tended to endorse (agree with) the item with a higher probability than members of another group with the same level of PSMU. Furthermore, the *problem* and *escape* items were most popular in the 15-year-old group but least popular in the 11-year-old group. Since we did not have any a priori hypotheses, we can provide only tentative explanations for these findings. For example, one of the reasons that *escape* was more popular for girls is that girls tend to use the coping strategy of social support more often (59). The fact that adolescents are able to use coping strategies more purposefully (60) might explain why the same item was more popular in older adolescents, as it speaks about the intentional use of social media to cope with negative feelings. In a similar vein, the 11-year-old group may be less aware that they are actually using social media to relieve themselves of negative emotions. However, more studies are needed to test if our tentative explanations are correct. Future studies may focus on the underlying motives and factors that affect specific symptoms (such as social connection, coping strategies, and emotional difficulties). In summary, our findings imply that some items function differently depending on age and gender. For example, we see the use of confirmatory factor analysis or item response theory models that can model partial measurement non-invariance between groups as more appropriate, since using raw scores, which implicitly assume invariance, could produce biased estimates and misleading results. In other words, although the non-invariance across gender and age groups in our study was only mild, it was not negligible. Therefore, ignoring it can bias the estimates of PSMU.

Furthermore, significant differences were found between boys and girls and between the younger and older age groups: the latent mean for girls was slightly higher than that for boys, and the latent means for 13- and 15-year-olds were slightly higher than for 11-year-olds. When both gender and age were considered simultaneously, there was no difference between 11-year-old boys and girls; however, a difference was observed between 13- and 15-year-old boys and girls with a higher latent mean for girls than for boys. Therefore, future studies should expect the interaction between age and gender and choose appropriate methods to capture it. In this regard, our findings conflict with some other studies (25, 29). In the original study (15), age did not have a significant effect, but boys showed more symptoms than girls. However, the authors tested the effects of gender and age separately and did not consider that the difference between boys and girls could vary depending on age.

Although we used the same research sample as Boer et al. (29), we extended the study in multiple ways. First, we analyzed and interpreted the local misfit of the unidimensional model, concluding that strict unidimensionality was not supported, although a single latent variable was a dominant source of item variance. Second, by estimating a residual network model, we identified small, but theoretically meaningful, residual relations between PSMU symptoms. Third, for measurement invariance analysis, we used more stringent guidelines designed for categorical data (61, 62). In doing so, we identified items whose thresholds slightly differed between gender and age groups. Furthermore, we interpreted the violations of measurement invariance in more detail, assessed their severity by comparing latent means estimated under different models, and assessed measurement invariance simultaneously for both gender and age. Additionally, we explored the construct validity of the SMDS using a structural model. As a result, the correlations among variables are not attenuated due to unreliability, since latent variables should not contain any error variance. Moreover, we can assess the fit of the whole model and assess potential cross-loadings or other unexpected effects. Lastly, we have extended the investigation of the construct validity of the SMDS by considering additional variables that should be associated with PSMU (e.g., preference for online interaction, gaming frequency, late sleep).

There are several limitations to our study that should be noted. First, only self-reported questionnaires were used, which could be affected by self-report bias, since adolescents themselves could misjudge the extent to which PSMU affects their lives. Future research may, therefore, consider the use of informant ratings (e.g., from parents), which can provide novel insights into the assessment of PSMU in adolescents, especially the “cognitive-affective” and “behavioral-consequential” aspects of PSMU. Second, although the present study used a large general sample of adolescents, future studies should verify the validity of the SMDS in clinical samples in order to test its ability to discriminate between clinical and non-clinical samples and its usefulness as a screening tool for the identification of PSMU. Third, although our results suggest that the mean level of PSMU symptoms may develop differently in boys and girls over time, due to the cross-sectional design of our study, it is not clear, whether this is truly an aging effect. In addition, due to the cross-sectional design of our study, we could not investigate predictive validity or test-retest reliability of the SMDS nor the temporal relationships between the symptoms. Therefore, longitudinal studies are needed to capture the developmental trajectory of PSMU in adolescents and to test measurement invariance and other psychometric properties of the SMDS over time. Fourth, since the data included only one scale that measured PSMU, we were unable to compare its psychometric properties with alternative scales. Fifth, the measures used in our study were not validated in the Czech language. However, the strict methodology of the HBSC survey was applied in the translation process. The quality of all translations was verified using the back translation method. The accuracy of the back translations were independently checked by a translation team within the HBSC network, which was specifically established for this task.

To conclude, the present study aimed to investigate the psychometric properties of the SMDS. Given its solid factor structure and good validity, we demonstrate its good psychometric properties and suggest that it is appropriate for the assessments of PSMU among adolescents. However, it should be noted that, even though support for a single dominant factor was found, strict unidimensionality was not achieved. Also, some violations of measurement invariance between gender and age groups, albeit small, were observed. Although we have suggested several explanations for these violations, they need to be tested by independent studies.

## Data availability statement

The raw data supporting the conclusions of this article will be made available by the authors, without undue reservation.

## Ethics statement

The study data collection was approved by the Institutional Research Ethics Committee of the Faculty of Physical Culture, Palacky University Olomouc, No. 9/2016, on 4 March 2016. Written informed consent was obtained from the individual(s) for the publication of any potentially identifiable images or data included in this article.

## Author contributions

NŠ, LB, and KR made the study concept, interpreted the results, edited the draft, and contributed to the writing of the paper. NŠ wrote the first draft. KR conducted the statistical analysis. All authors contributed to the article and approved the submitted version.

## Funding

This study was supported by Masaryk University (Project MUNI/A/1525/2021).

## Conflict of interest

The authors declare that the research was conducted in the absence of any commercial or financial relationships that could be construed as a potential conflict of interest.

## Publisher's note

All claims expressed in this article are solely those of the authors and do not necessarily represent those of their affiliated organizations, or those of the publisher, the editors and the reviewers. Any product that may be evaluated in this article, or claim that may be made by its manufacturer, is not guaranteed or endorsed by the publisher.

## References

[1] Allen KA Ryan T Gray DL McInerney DM Waters L. Social media use and social connectedness in adolescents: the positives and the potential pitfalls *Educ Dev Psychol*. (2014) 31:18–31. 10.1017/edp.2014.2

[2] Keles B McCrae N Grealish A. A systematic review: the influence of social media on depression, anxiety and psychological distress in adolescents. Int J Adolesc Youth. (2020) 25:79–93. 10.1080/02673843.2019.1590851

[3] Müller KW Dreier M Beutel ME Duven E Giralt S Wölfling K. A hidden type of internet addiction? Intense and addictive use of social networking sites in adolescents. Comput Hum Behav. (2016) 55:172–7. 10.1016/j.chb.2015.09.007

[4] Kuss DJ Griffiths MD. Online social networking and addiction—a review of the psychological literature. Int J Environ Res Public Health. (2011) 8:3528–52. 10.3390/ijerph8093528 22016701 PMC3194102

[5] American Psychiatric Association. Diagnostic and Statistical Manual of Mental Disorders (DSM-5). 5th ed. Washington, DC: American Psychiatric Association (2013).

[6] World Health Organization. International Statistical Classification of Diseases and Related Health Problems. 11th ed. Geneva: World Health Organization (2019).

[7] Valkenburg PM Meier A Beyens I. Social media use and its impact on adolescent mental health: an umbrella review of the evidence. Curr Opin Psychol. (2022) 44:58– 8. 10.1016/j.copsyc.2021.08.017 34563980

[8] Hussain Z Griffiths MD. The associations between problematic social networking site use and sleep quality, attention-deficit hyperactivity disorder, depression, anxiety and stress. Int J Ment Health Addiction. (2021) 19:686–700. 10.1007/s11469-019-00175-1

[9] Marino C Gini G Vieno A Spada MM. The associations between problematic Facebook use, psychological distress and well-being among adolescents and young adults: a systematic review and meta-analysis. J Affect Disord. (2018) 226:274–81. 10.1016/j.jad.2017.10.007 29024900

[10] Hussin Z Griffiths M.D. Problematic social networking site use and comorbid psychiatric disorders: a systematic review of recent large-scale studies. Front Psychiatry. (2018) 9:686. 10.3389/fpsyt.2018.00686 30618866 PMC6302102

[11] D'Arienzo MC Boursier V Griffiths MD. Addiction to social media and attachment styles: a systematic literature review. Int J Ment Health Addiction. (2019) 17:1094–118. 10.1007/s11469-019-00082-5

[12] Alonzo R Hussain J Stranges S Anderson KK. Interplay between social media use, sleep quality, and mental health in youth: a systematic review. Sleep Med Rev. (2021) 56. 101414. 10.1016/j.smrv.2020.101414 33385767

[13] Sun Y Zhang Y. A review of theories and models applied in studies of social media addiction and implications for future research. Addict behav. (2021) 114:106699. 10.1016/j.addbeh.2020.106699 33268185

[14] Brand M Rumpf H-Jü Demetrovics Z MÜller A Stark R King DL. Which conditions should be considered as disorders in the International Classification of Diseases (ICD-11) designation of “other specified disorders due to addictive behaviors”? J Behav Addict. (2020) 11:150–9. 10.1556/2006.2020.00035 32634114 PMC9295220

[15] van den Eijnden RJ Lemmens JS Valkenburg P.M. The social media disorder scale. Comput Hum Behav. (2016) 61:478–487. 10.1016/j.chb.2016.03.038

[16] Andreassen CS Torsheim T Brunborg GS Pallesen S. Development of a facebook addiction scale. Psychol Rep. (2012) 110:501–17. 10.2466/02.09.18.PR0.110.2.501-517 22662404

[17] Kircaburun K Griffiths MD. Instagram addiction and the Big Five of personality: The mediating role of self-liking. J Behav Addict. (2018) 7:158–70. 10.1556/2006.7.2018.15 29461086 PMC6035031

[18] Balakrishnan J Griffiths MD. An exploratory study of “Selfitis” and the development of the selfitis behavior scale. Int J Ment Health Addiction. (2017) 16:722–36. 10.1007/s11469-017-9844-x 29904329 PMC5986832

[19] Lou J Liu H Liu X. Development of the problematic social networking services use scale with college students. Soc Behav Pers. (2017) 45:1889–904. 10.2224/sbp.6179

[20] Esgi N. Development of social media addiction test (SMAT17). J Edu Train Stud. (2016) 4:174–81. 10.11114/jets.v4i10.1803

[21] Andreassen C Billieux J Griffiths M Kuss D Demetrovics Z Mazzoni E. The relationship between addictive use of social media and video games and symptoms of psychiatric disorders: a large-scale cross-sectional study. Psychol Addict Behav. (2016) 30:252–62. 10.1037/adb0000160 26999354

[22] Fung S. Cross-cultural validation of the Social Media Disorder scale. Psychol Res Behav Management. (2019) 12:683–90. 10.2147/PRBM.S216788 31695527 PMC6707349

[23] Dewi SY Lestari YM. Validity and reliability of indonesian social media disorder (SMD) scale in adolescent. J Profesi Medika. (2020) 14:2. 10.33533/jpm.v14i2.2049

[24] Afe T Ogunsemi O Ayotunde A Olufunke A Osalusi B Afe B. Psychometric properties and validation of the 9-item social media scale among pre-university students in Nigeria. East Asian Archi Psychiat. (2020) 30:108–12. 10.12809/eaap1946 33349617

[25] Boer M Stevens G Finkenauer C Ina HMK van den ER. Validation of the social media disorder scale in adolescents: findings from a large-scale nationally representative sample. Assessment. (2021). 10.31219/osf.io/2fphx. [Epub ahead of print]. 34189943 PMC9597146

[26] Sariçam H Adam Karduz FF. The adaptation of the Social Media Disorder scale to Turkish culture: validity and reliability study. J Meas Eval Educ Psychol. (2018) 9:116–35. 10.21031/epod.335607

[27] Savci M Ercengiz M Aysan F. Turkish adaptation of the social media disorder scale in adolescents. Noro Psikiyatr Ars. (2018) 55:248–55. 10.29399/npa.19285 30224872 PMC6138233

[28] Avcu A. Use of network psychometrics approach to examine social media disorder symptoms. ADDICTA Turkish J Addict. (2021) 8:87–91. 10.5152/ADDICTA.2021.20098

[29] Boer M van den Eijnden RJ Finkenauer C Boniel-Nissim M Marino C Inchley J. Cross-national validation of the Social Media Disorder-scale: Findings from adolescents from 44 countries. Addiction. (2022) 117:784–95. 10.1111/add.15709 34605094 PMC7614030

[30] Epskamp S Rhemtulla M Borsboom D. Generalized network psychometrics: Combining network and latent variable models. Psychometrika. (2017) 82:904–27. 10.1007/s11336-017-9557-x 28290111

[31] Kuss D Griffiths M. Social networking sites and addiction: ten lessons learned. Int J Environ Res Public Health. (2017) 14:311. 10.3390/ijerph14030311 28304359 PMC5369147

[32] Karacic S Oreskovic S. Internet addiction through the phase of adolescence: a questionnaire study. JMIR Ment Health. (2017) 4:e11. 10.2196/mental.5537 28373154 PMC5394260

[33] Inchley J Currie D Cosma A Samdal O. Health Behaviour in School-Aged Children (HBSC) Study Protocol: Background, Methodology and Mandatory Items for the 2017/18 Survey. St Andrews: Child and Adolescent Health Research Unit (CEHRU) University of St Andrews (2018).

[34] Gariepy G McKinnon B Sentenac M Elgar FJ. Validity and reliability of a brief symptom checklist to measure psychological health in school-aged children. Child Indicat Res. (2015) 9:471–84. 10.1007/s12187-015-9326-2

[35] World Health Organization. Well-being Measures in Primary Health Care/The Depcare Project. Copenhagen: WHO Regional Office for Europe (1998).

[36] Mascheroni G Ólafsson K. Different Media for Different Contacts. Net Child Go Mob Risks Oppor. 2nd ed. Milano: Educatt (2014).

[37] Livingstone S Haddon L Görzig A Ólafsson K. Risks and Safety on the Internet: The Perspective of European Children. Full Findings. London: EU Kids Online (2011).

[38] van Buuren S Groothuis-Oudshoorn K. Multivariate imputation by chained equations in R. J Stat Soft. (2011) 45:1–67. 10.18637/jss.v045.i03

[39] Rosseel Y. lavaan: An R package for structural equation modeling. J Stat Soft. (2012) 48:1–36. 10.18637/jss.v048.i0225601849

[40] Jorgensen TD Pornprasertmanit S Schoemann AM Rosseel Y. semTools: Useful Tools for Structural Equation Modeling. (2020). Available online at; https://CRAN.R-project.org/package=semTools (accessed March 15, 2022).

[41] Epskamp S. Psychonetrics: Structural Equation Modeling Confirmatory Network Analysis. (2021). Available online at: https://CRAN.R-project.org/package=psychonetrics.

[42] Rutkowski L Svetina D. Measurement invariance in international surveys: Categorical indicators & fit measure performance. Appl Meas Educ. (2017) 30:39–51. 10.1080/08957347.2016.1243540

[43] Svetina D Rutkowski L Rutkowski D. Multiple-group invariance with categorical outcomes using updated guidelines: An Illustration using Mplus and the lavaan/semTools packages. Struct Equ Model A Multidiscipl J. (2020) 27:111–30. 10.1080/10705511.2019.1602776

[44] Mansolf M Jorgensen TD Enders CK. A multiple imputation score test for model modification in structural equation models. Psychol Methods. (2020) 25:393–411. 10.1037/met0000243 31621350

[45] Li C-H. Statistical estimation of structural equation models with a mixture of continuous and categorical observed variables. Behav Research Methods. (2021) 53:2191–213. 10.3758/s13428-021-01547-z 33791955

[46] Haslbeck JM Epskamp S Marsman M Waldorp LJ. Interpreting the Ising model: the input matters. Multivar Behav Res. (2020) 56:303–13. 10.1080/00273171.2020.1730150 32162537

[47] Deserno MK Isvoranu AM Epskamp S Blanken T.F. Chapter 3. Descriptive analyses of network structures. In: Isvoranu AM Epskamp S Waldorp LJ Borsboom D, editors. Network Psychometrics With R: A Guide for Behavioral and Social Scientists. Routledge: Taylor & Francis Group (2022).

[48] Watson JC Prosek EA Giordano AL. Investigating psychometric properties of social media addiction measures among adolescents. J Counsel Dev. (2020) 98:458–66. 10.1002/jcad.1234727363464

[49] Borsboom D. A network theory of mental disorders. World Psychiatry. (2017) 16:5–13. 10.1002/wps.20375 28127906 PMC5269502

[50] Valkenburg PM Peter J. The differential susceptibility to media effects model. J Commun. (2013) 63:221–43. 10.1111/jcom.12024

[51] Petry NM Zajac K Ginley MK. Behavioral addictions as mental disorders: to be or not to be? Annu Rev Clin Psychol. (2018) 14:399–423. 10.1146/annurev-clinpsy-032816-045120 29734827 PMC5992581

[52] Kardefelt-Winther D Heeren A Schimmenti A van Rooij A Maurage P Carras M. How can we conceptualize behavioural addiction without pathologizing common behaviours? Addiction. (2017) 112:1709–15. 10.1111/add.13763 28198052 PMC5557689

[53] King DL Delfabbro PH. The cognitive psychology of Internet gaming disorder. Clinic Psychol Rev. (2014) 34:298–308. 10.1016/j.cpr.2014.03.006 24786896

[54] Orben A. Teenagers, screens and social media: a narrative review of reviews and key studies. Soc Psychiatry Psychiatr Epidemiol. (2020) 55:407–14. 10.1007/s00127-019-01825-4 31925481

[55] Kardefelt-Winther D. A conceptual and methodological critique of internet addiction research: towards a model of compensatory internet use. Comput Hum Behav. (2014) 31:351–4. 10.1016/j.chb.2013.10.059

[56] Starcevic V Billieux J. Does the construct of Internet addiction reflect a single entity or a spectrum of disorders? Clin Neuropsychiatry. (2017) 14:5–10.

[57] Papini S Rubin M Telch MJ Smits JA Hien D. A Pretreatment posttraumatic stress disorder symptom network metrics predict the strength of the association between node change and network change during treatment. J Trauma Stress. (2020) 33:64–71. 10.1002/jts.22379 31343789

[58] Jones PJ Ma R McNally RJ. Bridge centrality: a network approach to understanding comorbidity. Multivar Behav Res. (2021) 56:353–67. 10.1080/00273171.2019.1614898 31179765

[59] Eschenbeck H Kohlmann C-W Lohaus A. Gender differences in coping strategies in children and adolescents. J Individ Differ. (2007) 28:18–26. 10.1027/1614-0001.28.1.18

[60] Zimmer-Gembeck MJ Skinner E. A Review: The development of coping across childhood and adolescence: an integrative review and critique of research. Int J Behav Dev. (2011) 35:1–17. 10.1177/0165025410384923

[61] Chen FF. Sensitivity of goodness of fit indexes to lack of measurement invariance. Struct Equ Model A Multidisciplinar J. (2007) 14:464–504. 10.1080/10705510701301834

[62] Cheung GW Rensvold RB. Evaluating goodness-of-git indexes for testing measurement invariance. Struct Equ Model A Multidisciplinar J. (2002) 9:233–55. 10.1207/S15328007SEM0902_522551991

